# Editorial: Cancer genomics in the era of precision medicine

**DOI:** 10.3389/fgene.2024.1378917

**Published:** 2024-02-16

**Authors:** Omar M. Khan, Domenico Mallardo, Julie Decock

**Affiliations:** ^1^ College of Health and Life Sciences (CHLS), Hamad Bin Khalifa University (HBKU), Qatar Foundation (QF), Doha, Qatar; ^2^ Department of Melanoma, Cancer Immunotherapy and Development Therapeutics INT IRCCS Fondazione “G Pascale”, Napoli, Italy; ^3^ Cancer Research Center, Qatar Biomedical Research Institute (QBRI), Hamad Bin Khalifa University (HBKU), Qatar Foundation (QF), Doha, Qatar

**Keywords:** cancer genomics, precision medicine, epigenetics, biomarkers, diagnostics, predictive value

Cancer is a multifactorial disease driven by genetic and environmental factors, affecting a range of biological processes which regulate cell proliferation and survival. Advancements in sequencing technology and big data analysis have made genomics a key pillar of cancer research, providing insights into disease etiology, disease risk and treatment resistance mechanisms. In the clinical setting, cancer genomics is currently used to determine hereditary cancer risk, predict prognosis, and inform treatment selection. In breast cancer, for example, several prognostic and predictive genomic tests have been developed to predict the risk of recurrence and likely benefit of chemotherapy (Chowdhury et al., 2023). Furthermore, comprehensive genomic profiling of tumors helps to identify actionable mutations that can be targeted with specific drugs or therapies. For instance, melanoma patients whose tumors test positive for the BRAF V600E mutation by a companion diagnostic test were shown to specifically benefit from treatment with BRAF and MEK inhibitors alone or in combination with immune checkpoint inhibitors (Cheng et al., 2012; Dummer et al., 2023; Ascierto et al., 2024).

Interestingly, cancer genomics can also predict the outcome of revolutionary immune-based therapies. In stage III melanoma, patients with a high IFN-γ related gene expression score exhibit a better response to immunotherapy, highlighting the value of this signature as a promising predictive biomarker (Rozeman et al., 2021). Cancer patients with mismatch repair (MMR) pathway mutations were found to be more likely to respond to PD-1 immune checkpoint inhibitors, indicating that testing for MMR deficiency could be used as a predictive biomarker in solid tumors (Le et al., 2017; Chalabi et al., 2020). Thus, cancer genomics can be used to guide clinical decisions in precision medicine and can provide a greater opportunity for success in difficult-to-treat cancers using personalized treatment approaches.

Genomic features and chromosomal abnormalities are also used to classify acute myeloid leukemia (AML) patients into favorable-, intermediate- and adverse-risk groups. However, this classification does not fully recapitulate the biological heterogeneity within each group. In particular, patients within the intermediate risk group exhibit diverse clinical outcomes, highlighting the need for new molecular signatures that can help to improve stratification of intermediate-risk patients. To address this shortcoming, Eshibona et al. investigated gene expression profiles of 447 AML patients. Patients were first categorized into a short survival (<365 days, SS) and long survival (>3,650 days, LS) group which were further stratified into two risk subcategories each based on cytogenetic risk: poor and intermediate-poor for SS and good and intermediate-good for LS. Differential expression analysis of the SS and LS groups identified 87 differentially expressed genes of which nine were significantly associated with worse prognosis with AUC values ranging from 0.69 to 0.84. Furthermore, expression of all nine genes was significantly different between patients in the intermediate-poor and intermediate-good subgroups. In addition, the expression of four out of nine genes (CD109, CPNE3, DDIT4, and INPP4B) did not differ within the SS subgroups (poor and intermediate-poor) or within the LS subgroups (good and intermediate-good), indicating that their expression may provide a more accurate stratification of intermediate-risk patients into SS and LS risk groups compared to cytogenetic classification. Similarly, Li T et al. developed a prognostic gene signature to predict the clinical outcome of multiple myeloma patients. Specifically, the authors focused on oxidative stress and cuproptosis related genes as mediators of tumorigenesis. First, they generated a co-expression network of cuproptosis-related genes and oxidative stress genes, resulting in the identification of 419 cuproptosis-related oxidative stress genes of which 76 were differentially expressed in multiple myeloma samples compared to healthy controls. Of the 76 genes, 26 were significantly associated with prognosis and eight were used to generate a prognostic risk model. Validation of the 8-gene signature in more than 2,000 patients demonstrated that the overall survival of patients with a high-risk score was significantly shorter than of patients with a low-risk score. Li J et al. established a cellular senescence-related gene signature to predict the clinical outcome of patients with hepatocellular carcinoma. Differential expression analysis of the TCGA database identified 70 cellular senescence-related genes to be dysregulated in patients with hepatocellular carcinoma of which 25 were associated with prognosis. Finally, using LASSO Cox regression analysis, four genes (EZH2, G6PD, CBX8, or NDRG1) were selected to construct a prognostic risk signature with an AUC of 0.687 at 5 years. Comparative analysis revealed a higher mutational burden in the high-risk group as well as differences in drug sensitivity, the abundance of immune cell subsets and expression of immune checkpoints. Finally, Wang L et al. investigated the presence of genomic alterations and changes in expression of the CTC1-STN1-TEN1 (CST) complex across 33 cancer types from the TCGA database. This complex plays an important role in the regulation of telomere replication and genome stability and has been shown to enhance treatment response to radiotherapy and PARP inhibition. Somatic alteration analysis found that CTC1/STN1 deletion and mutations together with TEN1 amplifications were the most common alterations. Overall, expression of CSC1 and STN1 was reduced in tumor tissues compared to adjacent normal tissues while the opposite was true for TEN1. Consensus clustering revealed three clusters whereby patients with high tumor expression of CTC1 and STN1 and low expression of TEN1 showed the best survival, and the lowest telomerase activity, cell proliferation rate, and genome instability. Furthermore, CTC1 was found to be a negative regulator of c-MYC through pathway analysis and knockdown experiments, and CTC1-STN1 were positively associated with better response to immunotherapy. Several chemical compounds were predicted to modulate CST expression, providing further rationale for functional and therapeutic studies of the complex in cancer.

In addition to genetic mutations and alterations, tumor cells can also undergo epigenetic modifications, resulting in gene expression changes that facilitate tumorigenesis. N6-methyladenosine (m6A) modification is the most abundant epigenetic alteration in mRNAs, non-coding RNAs and ribosomal RNA which impacts the regulation of RNA processing, splicing, nucleation, translation, and stability. Here, Wan et al. used bioinformatic and machine learning approaches to identify m6A-related lncRNA signatures with prognostic significance in gastric cancer patients. Analysis of the respective TCGA dataset identified a total of 697 differentially expressed m6A-related lncRNAs of which 18 were associated with clinical outcome. Cox regression analysis identified 11 m6A-lncRNAs as the most significant prognostic markers which were subsequently used to generate a prognostic risk score or m6A-related lncRNA prognostic signature (m6A-LPS). Within the m6A-LPS, 2 lncRNAs (AL512506.1 and AL391152.1) were found to form a competing endogenous RNA network of 7 miRNAs and 90 target mRNAs that were enriched in biological processes such as cell cycle, cell division and signaling pathways. Biological validation in the gastric cancer cell line SGC-7901 demonstrated a downregulation in cell cycle regulator expression and reduction in the number of cells in G2/M phase following depletion of AL391152.1.

To conclude, this Research Topic of articles illustrates how cancer genomics can provide novel insights into cancer biology at a population and individual level and can help guide crucial decisions about treatment and care through a more personalized approach or precision medicine.
